# Denture base adaptation, retention, and mechanical properties of BioHPP versus nano-alumina-modified polyamide resins

**DOI:** 10.34172/joddd.2021.039

**Published:** 2021-12-05

**Authors:** Radwa Mohsen Kamal Emera, Reham Mohammed Abdallah

**Affiliations:** ^1^Prosthodontics Department, Faculty of Dentistry, Mansoura University, Mansoura, Egypt; ^2^Dental Biomaterials Department, Faculty of Dentistry, Mansoura University, Mansoura, Egypt; ^3^Dental Biomaterials Department, Faculty of Dentistry, Horus University, New Damietta, Egypt

**Keywords:** Adaptation, Al_2_O_3_ NPs, BioHPP, Flexural strength, Hardness, Retention

## Abstract

**Background.** Continuous development of denture base materials has led to the introduction of innovative alternatives to polymethyl methacrylate. The present study aimed to evaluate the mechanical properties, adaptation, and retention of alumina nanoparticles (Al_2_O_3_ NPs) modified polyamide resin versus BioHPP (high-performance polymer) denture base materials.

**Methods.** Four groups of specimens, one control (group I) (unmodified polyamide) and two groups (groups II and III) (2.5 and 5 wt% Al_2_O_3_ NP-modified polyamide, respectively) versus BioHPP specimen group (group IV), were tested for surface microhardness and flexural strength. Complete dentures fabricated from 5 wt% Al_2_O_3_ NP-modified polyamide resin and BioHPP were used to evaluate denture base adaptation and retention.

**Results.** The higher concentration in the alumina NP-modified polyamide group (5 wt%) demonstrated significantly higher flexural strength values and insignificantly higher hardness values than the lower concentration (2.5 wt%). There was a significant increase in the BioHPP group in both flexural strength and surface hardness compared to all polyamide groups. A statistically insignificant difference was observed between the two denture base materials regarding mean misfit values of the calculated total tissue surface area and four of the total seven evaluated areas. Satisfactory and comparable retention values were observed for both denture base materials.

**Conclusion.** BioHPP and Al_2_O_3_ NP-modified polyamide resin could be used as a promising alternative denture base material with good adaptation, retention, and mechanical properties.

## Introduction

Complete dentures are considered the most cost-effective and least invasive option for edentulous patients’ rehabilitation.^1^ A vital feature affecting the excellence of removable prostheses is the denture fit. Well-fitting dentures offer more comfort and decrease the incidence of traumatic ulcers.^2^ Tissue matching denture fit is crucial for adequate complete dentures retention that affects phonetics and masticatory efficiency.^3^ Consequently, obtaining the greatest tissue fit should be one of the principal targets in constructing a complete denture.^4^

Polymethyl methacrylate (PMMA) resin is the most commonly used material for denture base fabrication because of its good mechanical properties, aesthetics, tissue compatibility, and easy repair. However, dimensional changes after processing are considered a significant drawback.^5^ Dimensional changes of denture base can occur due to curing shrinkage and expansion, thermal shrinkage, water absorption, and internal stress release.^6^ This change can cause poor denture-tissue adaptation and compromise denture retention and stability.^7^

One method to overcome the mechanical deficiencies of PMMA is to use alternative polymers such as polyamides (nylon plastics).^8^ Nylon is a crystalline polymer, while PMMA is amorphous. This crystalline structure accounts for the lack of nylon solubility in solvents, in addition to the high heat resistance, good strength, and ductility.^9^ Furthermore, nylon materials have other attractive advantages, including safe toxicity for patients with resin monomer and metal allergy, higher elasticity, and use of heat-molding rather than chemical polymerization to control the polymerization shrinkage and its associated deformation.^10^ However, polyamides need modification to achieve more favorable characteristics than the present PMMA materials.^11^

Al_2_O_3_ ceramic fillers were used for reinforcement of acrylic resin. These fillers have lower density; hence, the acrylic resin’s lightweight is maintained. As the ceramic particles are white, the possibility of the color change of the denture base is low.^12^ Arora et al^13^ reported a positive influence of adding alumina particles to acrylic resins. It enhanced resin thermal conductivity, increasing patient satisfaction. Additionally, the impact strength, flexural strength, tensile strength, compressive strength, and surface hardness improved.^14^

Poly-ether-ether-ketone (PEEK), a semi-crystalline plastic, can be considered an innovative material to replace PMMA because of its good mechanical, chemical, and physical properties.^15^ BioHPP (high-performance polymer) is a member of the PEEK family containing 20% ceramic fillers. BioHPP is a favorable prosthetic restoration with excellent stability, low plaque affinity, and optimum polishing properties. Its modulus of elasticity is close to the human bone; thus, it improves the transmission of masticatory forces. Therefore, it can be applied for the construction of removable dentures and obturators.^16^

Although adaptation, retention, and mechanical properties of different denture base materials are clinically important, only a few studies are available on the Al_2_O_3_ NPs modified polyamide versus BioHPP materials. Consequently, this study was conducted to clarify the mechanical properties, adaptation, and retention of these attractive modern denture base materials.

## Methods

In this study, two types of thermoplastic resins were used to fabricate specimens used for both flexural strength and surface hardness tests and fabrication of complete denture bases for adaptation and retention tests. The first thermoplastic resin was polyamide resin (Dentiflex; Roko, Poland) modified by adding two concentrations of Al_2_O_3_ NPs (2.5 and 5 wt%). The second resin was BioHPP high-performance polymer (BioHPP, Bredent GmbH & Co., Germany).

Forty specimens were used for both flexural strength and surface hardness tests (20 specimens for each test). In each test, the groups were divided as follows (n = 5):

- Group I: Unmodified polyamide specimens (control)
- Group II: 2.5 wt% alumina NP-modified polyamide specimens
- Group III: 5 wt% alumina NP-modified polyamide specimens
- Group IV: BioHPP specimens

### Specimen preparation

#### Polyamide specimen preparation

For polyamide specimen preparation,five cartridges of polyamide were emptied from their content of granules. Then, the cartridges were weighed using an electronic balance, and the average weight of the five empty cartridges was recorded. Five full polyamide cartridges were weighed, and the average weight was registered. Therefore, the weight of the granules inside the cartridge was recorded by subtracting the average weight of full cartridges from that of empty ones. In the current study, Al_2_O_3_ nanoparticle powder (Sigma-Aldrich CO., St. Louis, MO, USA) with a particle size of <50 nm by transmission electron microscope (TEM) was used. The nanoparticle powder representing 2.5 and 5 wt% of the weight of the polyamide granules was weighed and placed inside a plastic tube.

The polyamide was placed on a vibrator and shaken very well to ensure the uniform distribution of the added nanoparticle powder into the polyamide cartridge. The furnace was set to 260ºC for heating the cylinder. The heating cylinder was then removed from the furnace after melting the cartridge. Before injecting the material into the flask, Teflon rectangles and disks were invested in the flasks filled with dental stone. The flasks were opened, and the Teflon rectangles and desks were removed after the stone setting, forming rectangular and disk-shaped cavities in the stone, used as matrices to create polyamide resin specimens.

The flask was placed in a thermopress injection molding unit. The resin was injected into the flask using 5-bar pressure. Finally, the flask was removed from the injection unit after releasing the pressure and left for bench cooling to room temperature. The specimens were finished and polished with rubber wheels on the mandrills after being removed from the flask. They were kept for 48 hours in distilled water at 37 ± 1ºC before testing.

### BioHPP specimen preparation

BioHPP specimens werefabricated using the compression molding technique at 400ºC under a load of 60 MPa.

### Flexural strength test

Bar-shaped specimens measuring 65×10×3 mm were prepared for flexural strength testing according to American Dental Association (ADA) Speciﬁcation No. 12^17^ using a universal testing machine (Instron 8871, Instron Co.). Each specimen was supported at both ends with 50-mm spans. A 490-N load cell was applied at the center of the opposing surface at 5 mm/min crosshead speed. The maximum load applied on the specimens was recorded, and the ﬂexural strength of the specimen was calculated using the equation: S = 3WL/2bd^2^

where S is the ﬂexural strength in MPa, W is the maximum load applied to the specimen in Newton, L is the support span (50 mm), b is the specimen width (10 mm), and d is the thickness (2.5 mm).

### Surface hardness test

Disk specimens measuring 2 mm in thickness and 40 mm in diameter were prepared for surface hardness testing. A microhardness tester (Model MHT-1, No. 8621, Matsuzawa Seiki Co., LTD., Tokyo, Japan) was used with a square-based pyramid indenter and a load of 300 g applied at 15-s dwell time. Three indentations were made at different points of each specimen surface, and an average value was calculated.

### Fabrication of complete dentures for evaluating denture base adaptation and denture retention

Ten healthy, completely edentulous patients were selected for complete denture construction. The residual alveolar ridges of the maxilla and mandible were healthy, firm, and free from any signs of inflammation or ulceration. All the patients had a normal maxillo-mandibular relationship. All the participants were informed of the study treatment plan and signed informed consent forms. The study protocol was approved by the institutional ethics committee.

Polyamide resin used to fabricate maxillary dentures was modified by adding Al_2_O_3_ NPs (5 wt%) because it is the percentage offering the most promising in vitro results regarding the material physical properties. Thus, for each patient, a mandibular heat-cured acrylic resin complete denture was constructed against two maxillary complete dentures (5 wt% Al_2_O_3_ NP-modified polyamide resin and BioHPP) through the following steps:

Record blocks were constructed over the master casts, and maxillo-mandibular relation was recorded. The maxillary cast was mounted using facebow transfer, while the lower cast was mounted after recording the centric relationship. Setting up of the suitable-sized artificial acrylic teeth was carried out. The trial dentures were then tried in the patient’s mouth. The mandibular waxed-up trial denture was flasked, and the heat-cured acrylic resin was packed and cured using the long curing cycle.

**Figure 1:**
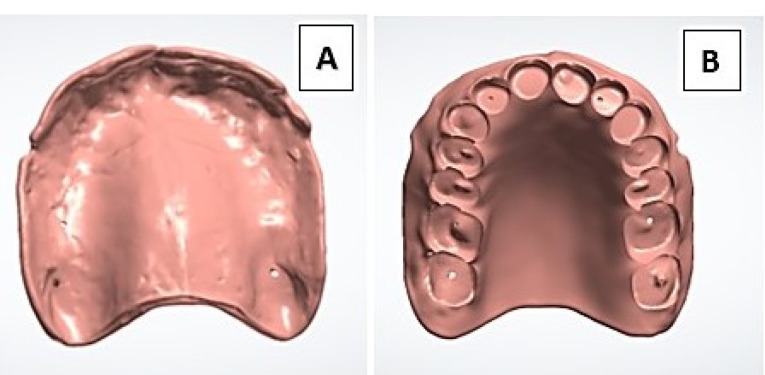
Final virtual design of maxillary denture base. A: Fitting surface and denture border. B: Polished surface showing recesses prepared for artificial teeth.

The maxillary master cast and the maxillary trial denture were scanned using a 3D scanner (Swing 3D Dental Scanner, Korea) to start the designing process of the maxillary denture base using the CAD software (EXO CAD Software, Dental DB 2.2 Valletta, version 2.2 Engine Build 6654). The design included prepared recesses that accurately fit each denture tooth as guided by the 3D image of scanned trial dentures (Figure 1).

**Figure 2:**
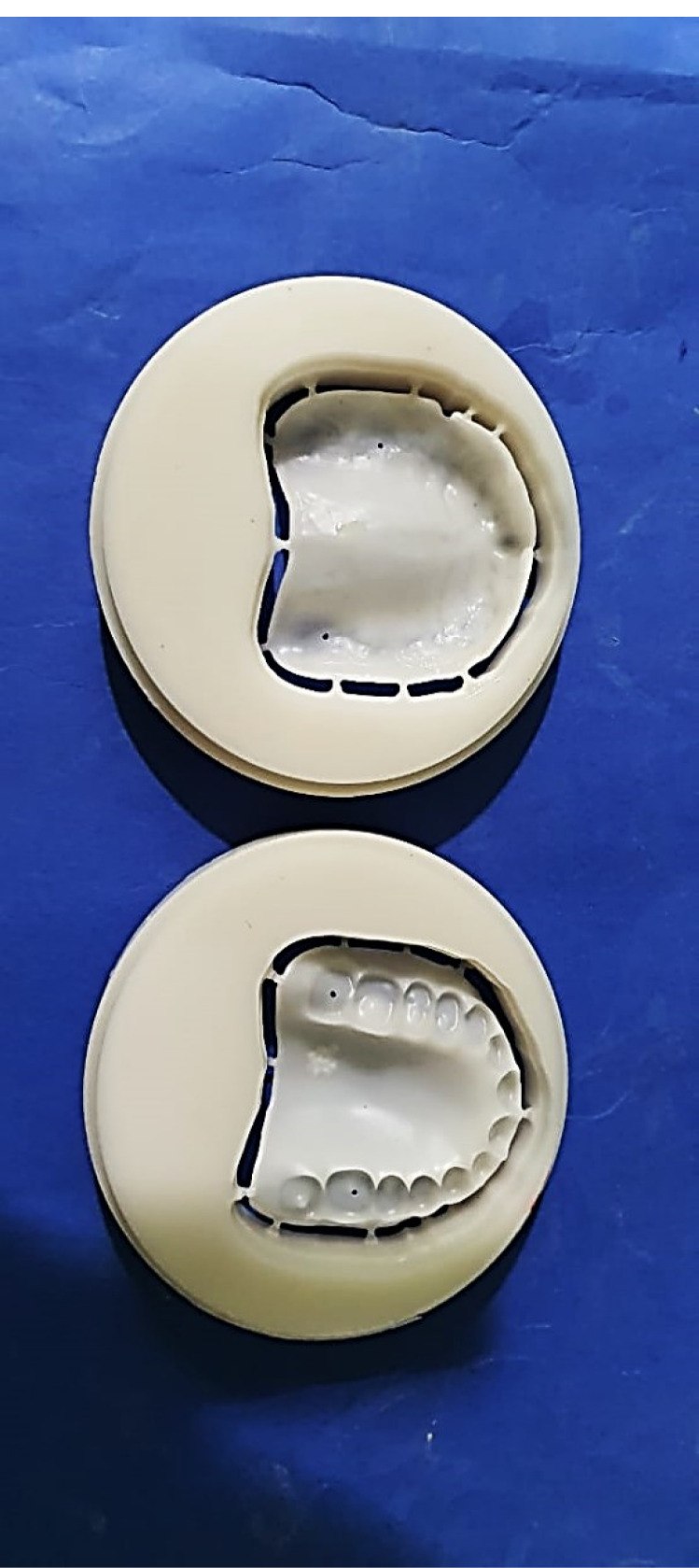
Milled maxillary denture bases of dental CAD-CAM milling wax.

**Figure 3:**
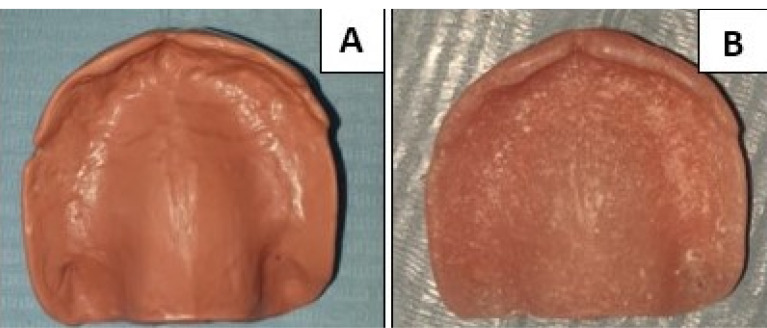
Finished maxillary dentures. A: BioHPP denture base. B: Al_2_O_3_ NP-modified polyamide denture base.

STL (standard tessellation language) file format of the final denture base design was imported to the milling machine (MILL Box 2018 Milling Machine: ARUM, 400 Corea) for milling two identical maxillary denture bases of dental CAD-CAM milling wax (Luoyang Penghao Ceramic Technology Co., Ltd) (Figure 2). One wax denture base was replaced by polyamide resin (Dentiflex; Roko, Poland) modified by adding Al_2_O_3_ NPs (5 wt%) using a thermopress injection molding unit. The second one was replaced with BioHPP (BioHPP, Bredent GmbH & Co., Germany) using the compression molding technique (Figure 3). Previously selected maxillary artificial teeth were cemented to each final denture base with a bonding agent (Visio Lign, Bredent). Laboratory remount was carried out, and the needed occlusal adjustments were performed. At insertion appointment, proper denture base fit, border extension, and premature occlusal contacts were checked.

### Evaluation of denture base adaptation

The denture base adaptation was evaluated; then, the patients were left to function with each denture set for one week, one month, and three months, respectively, during which they were recalled to test denture retention. To eliminate the effect of neuromuscular adaptation of the patient on denture retention, the patients were randomly divided into two equal groups. One group received maxillary polyamide denture at first, which was replaced by a BioHPP one after three months, with the reverse order in the other group.

To evaluate denture base adaptation, the fitting surfaces of the denture bases were coated with a scanning spray (EZ Scan, AlphaDent), and the denture bases were mounted on a silicone positioning index (Exaflex Putty, GC Corp) to confirm the same position and angulation for each denture during scanning (Swing 3D Dental Scanner, Korea). The obtained scanned 3D image was exported to an STL file. The STL file for each denture’s fitting surface was superimposed on the STL file of the corresponding master cast using surface matching software (Geomagic Verify; 3D Systems).^5,18^

**Figure 4:**
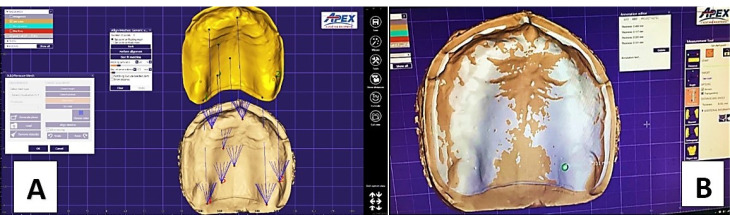
Evaluation of denture base adaptation. A: Superimposition of STL file of the scanned maxillary master cast and scanned denture base. B: Created color map of denture surface adaptation.

Color surface maps were obtained for visual displaying of the denture base adaptation to the cast. Fit discrepancies were evaluated by computing the distance between the two superimpositions (Figure 4). The analyses were performed for the whole fitting surface and specific regions of interest: anterior ridge, posterior crest, vestibular flange, palatal vault, posterior palatal seal area (PPS), mid-palatal raphae, and tuberosity to assess the region-specific mismatches.^19^

### Evaluation of denture retention

Maxillary denture retention was evaluated for each denture base material after one week (T0), one month (T1), and three months (T3) of denture function. Forcemeter device was used to measure the retention in a vertical direction perpendicular to the patient’s occlusal plane.^20^

**Figure 5:**
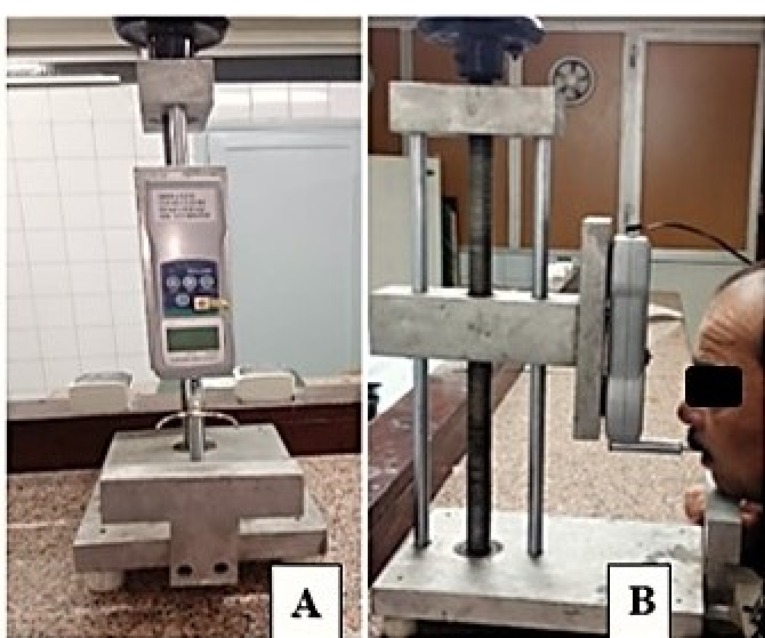
Measuring maxillary denture retention. A: Digital forcemeter fixed to the measuring device. B: The patient resting his chin on the chin support of the device.

Four hooks were attached at the buccal flange at the canine and first molar areas at the same height using auto-polymerized acrylic resin. The maxillary denture was completely seated in the patient’s mouth. The patient was asked to rest his chin on the device chin support, keeping the mandibular occlusal plane parallel to the floor (Figure 5). The hooks would engage intraorally to the fork of the force meter at the pull end. The force gauge was used to measure the pull force needed to dislodge the maxillary denture from its place. Five readings were recorded, and the average was calculated.

## Results

All the statistical analyses were performed with SPSS (IBM SPSS Statistics, version 22.0; IBM Corp) (α = 0.05). Shapiro-Wilk test was used to examine the normal distribution of data. All the data for both flexural strength and surface hardness tests were analyzed using two-way ANOVA and post hoc Tukey analysis with a significant factor of α = 0.05. Comparison between misfit and retention values of the two materials was performed using independent samples t-test.

The mean and standard deviation values for flexural strength and surface microhardness are presented in Table 1.

**Table 1:** Means (standard deviations) of mechanical properties of BioHPP and polyamide with Al_2_O_3_ NPs incorporation and Tukey analysis

**Group**	**Hardness test (kg/mm** ^2^ **)**	**Flexural strength test (MPa)**
Group I: Polyamide (control)	15.850^c^ (0.473)	79.000^d^ (0.183)
Group II: Polyamide (2.5 wt% Al_2_O_3_ NPs)	20.325^b^ (1.021)	90.450^c^ (1.323)
Group III: Polyamide (5 wt% Al_2_O_3_ NPs)	21.100^b^ (1.347)	99.000^b^ (1.344)
Group IV: BioHPP	24.050^a^ (0.881)	114.800^a^ (0.839)
*P* value	0.0001	0.0001

Mean values for each property represented with the same superscript letter (column) are not significantly different (*P*≥0.05), while the mean values with different letters are significantly different (*P* < 0.05).

### Hardness test

BioHPP exhibited the highest mean hardness values compared to all groups of polyamide (control and experimental groups). Moreover, the differences between all the groups were significant (*P* < 0.05). There was a significant increase in the mean hardness values of both 2.5 and 5 wt% Al_2_O_3_ NP-modified polyamide groups compared to that of the polyamide control group. The higher concentration of Al_2_O_3_ NPs (5 wt%) exhibited higher mean hardness values than the 2.5 wt% group, although the difference was not significant.

### Flexural strength test

Regarding mean flexural strength values,BioHPP exhibited the highest value in comparison to the control and experimental polyamide groups. The group of polyamide modified with 5 wt% Al_2_O_3_ NPs showed the highest mean flexural strength values in comparison to the control and 2.5 wt% Al_2_O_3_ NP-modified polyamide groups. Meanwhile, the control group exhibited the lowest values. The difference between the control and experimental polyamide groups was significant.

### Denture base adaptation

Mean misfit values (mismatching between the denture base intaglio surface and the master cast) are presented in Table 2. A statistically significant difference was observed between the two denture base materials regarding mean misfit values of the three evaluated areas (median palatine raphe, vestibular flange, PPS). However, the difference was insignificant regarding the values of the four remaining areas evaluated (anterior ridge, palatal vault, posterior crest, and tuberosity) and the calculatedtotal tissue surface area.

**Table 2:** Descriptive statistics of misfit values for both materials in millimeters

**Area**	**BioHPP** **(M±SD)**	**Reinforced polyamide** **(M±SD)**	**Independent-samples** ***t*** ** test**
Anterior ridge	0.039 ± 0.012	0.040 ± 0.009	0.795
Palatal vault	0.021 ± 0.007	0.019 ± 0.008	0.636
Median palatine raphe	0.027 ± 0.002	0.057 ± 0.015	0.000*
Posterior crest	0.057 ± 0.017	0.072 ± 0.030	0.248
Vestibular flange	0.175 ± 0.027	0.232 ± 0.158	0.001*
Tuberosity	0.128 ± 0.027	0.151 ± 0.030	0.137
PPS	0.055 ± 0.002	0.109 ± 0.007	0.000*
Total	0.058 ± 0.019	0.072 ± 0.042	0.069

M, mean; SD, standard deviation; PPS, posterior palatal seal. *Significant.

### Retention

Tables 3 and 4 present the mean retention force values of both denture base materials at different follow-up periods. Satisfactory and comparable retention values were observed for both denture base materials, with statistically insignificant differences between them at all follow-up periods.General linear model test for repeated measures showed a significant difference in retention values between different follow-up periods within each material group. Multiple comparisons between two follow-up periods using paired-samples *t* test revealed a significant difference in retention values one week after denture insertion and one month and three months later, while the difference between retention values one month after denture insertion and after three months was insignificant for both materials.

**Table 3:** Mean retention force values of both materials at different follow-up periods

**Follow-up time**	**BioHPP** **(M±SD)**	**Reinforced polyamide** **(M±SD)**	**Independent-samples** ***t*** ** test**
T0	51.607 ± 6.849	50.767 ± 8.214	0.828
T1	53.893 ± 6.677	52.310 ± 7.436	0.661
T3	54.037 ± 6.649	52.846 ± 7.158	0.735
General linear model (Repeated measures)	0.001*	0.022*	

M, mean; SD, standard deviation; T0, one week after denture insertion; T1, one month after denture insertion; T2, three months after denture insertion. *Significant.

**Table 4:** Multiple comparisons of retention values between each two follow up periods for both materials

	**P1**	**P2**	**P3**
BioHPP	0.001*	0.000*	0.194
Reinforced polyamide	0.029*	0.014*	0.135

Each cell showing the *P* value of paired-samples *t* tests where: P1: Comparison of retention one week afterdenture insertion and one month later. P2: Comparison of retention one week after denture insertion and three months later. P3: Comparison of retention one month after denture insertion and after three months. * Significant

## Discussion

According to the results of this study, PEEK demonstrated the highest hardness and flexural strength values compared to all groups of polyamide resin, which could be explained because PEEK’s mechanical properties are comparable to those of dentin and enamel. Hence, PEEK could have an advantage over ceramic structures like alumina used in the modification of polyamide.^21^ This finding was supported by Zok and Miserez,^22^ who reported that although PEEK has significantly low elastic modulus, its abrasive resistance, hardness, and flexural strength are similar to metallic alloys.

Moreover, PEEK is a two-phase, semi-crystalline polymer, with 30%‒35% crystallinity, according to its manufacturing process. Since the degree of crystallinity has a considerable effect on the mechanical properties, the higher the crystalline packing rate, the harder and more brittle the material will be. Consequently, this clarifies why PEEK exhibited significantly higher hardness values than all polyamide groups. Additionally, modification of PEEK with 20% ceramic fillers to develop BioHPP and improve PEEK’s properties greatly enhances its hardness and flexural strength properties.^23^

Regarding polyamide resin, its modification with either 2.5 or 5 wt% Al_2_O_3_ NPs exhibited a significant increase in its surface hardness and flexural strength values compared to its control group. These results could be related to the fact that the Al_2_O_3_ NPs might serve as fillers in the polyamide polymer, which enhanced its thermal diffusivity because it has low thermal conductivity and diffusivity.^24^ This finding is consistent with another study demonstrating that polymer thermal conductivity was improved by adding thermally conductive fillers, metal particles, or ceramics.^25,26^ Nanoparticles can be used as additives owing to their unique size-dependent properties.^27^ In the current study, alumina nano-sized additives might enhance the thermal diffusivity of polyamide denture base material. Thus, its melting and injection procedure might be enhanced.

The particle size of Al_2_O_3_ NPs impacts the distance between particles, affecting the thermal conductivity, diffusivity, and mechanical properties of the polyamide resin. The distance between particles might be smaller because of the fillers’ very small size,^28^ resulting in paths or bridges with enhanced thermal diffusivity and conductivity, hence, the mechanical properties of polyamide modified groups.

Another explanation for these results is that adding nano-sized additives to polyamide denture base materials might influence its crystallinity that could affect its mechanical properties, increasing its surface hardness and flexural strength, particularly with the higher concentration of Al_2_O_3_ NPs (5 wt%).^24^

The increase in flexural strength of Al_2_O_3_ NP-modified polyamide could be clarified on a transformation toughening basis. Al_2_O_3_ NPs return to the most stable hexagonal alpha phase at high temperatures. This is the phase of certain significance for structural applications.^17^ When adequate stress progresses and microcracks start to spread, the transformation phenomenon happens, exhausting the energy for crack propagation.^29^ Thus, the appropriate distribution of the filler inside the matrix can stop or deflect cracks.^30^

Moreover, the hardness significantly increased after adding 2.5 and 5 wt% of Al_2_O_3_ NPs to polyamide. This finding agrees with Abdel Samad and El Fallal,^31^ who found that reinforcing acrylic resin with ceramic particles can yield some enhancements in the surface hardness. This increase in hardness might be attributed to strong ionic interatomic bonding of Al_2_O_3_ NPs, producing its required material properties, i.e., hardness and strength. The most stable hexagonal alpha phase Al_2_O_3_ NPs is the strongest and stiffest of the oxide ceramics. Hence, when Al_2_O_3_ NPs diffuse in a matrix, they enhance its hardness and strength.^17^

Fit, which means the match between the denture base intaglio surface and the master cast, is one of the most significant criteria for assessing prostheses and directly affects maxillary complete dentures retention.^32,33^ Misfit measurement using analysis of the superimposed image of scanned denture base and the master cast has been used for investigating denture base adaptation. The entire fitting surface of the denture base was assessed as an alternative method of using geometric reference points for surface matching.^4^

Dimensional stability was digitally evaluated in the present study using a surface matching program and scanning device. However, previous studies used an optical microscope to measure the distance between landmark points of dentures, and some of them used simple calipers. These methods were restricted to the overall deformation because measuring the two points was only a linear analysis.^34^

There were insignificant differences between the two studied materials regarding misfit values of the calculatedtotal tissue surface area. Measurements were small, ranging between 0.058 mm for BioHPP and 0.072 mm for reinforced polyamide, which might be acceptable in a clinical context as concluded by Goodacre et al.^33^ This finding might be explained by the superior physical properties of BioHPP and Al_2_O_3_ NP-modified polyamide resin. It was concluded that adding Al_2_O_3_NPs to PMMA increases its thermal stability (reducing the thermal expansion coefficient and contraction) and flexural strength and, at the same time, decreases water sorption and solubility.^6^ Moreover, the accuracy of the milled denture base wax patterns offered more precise fitting of the final denture bases.^35^

The first evaluation period of maxillary denture retention was after one week of denture insertion. This is because the denture needs about one week to allow its settlement and adaptability to the underlying tissues to produce sufficient retention.^36^

The acceptable and comparable retention values observed for both groups might be attributed to the recorded satisfactory adaptation of denture bases.^37^ Multiple comparisons between each two follow-up period revealed a significant difference in retention values. In contrast, the difference between retention after one month and three months of denture insertion was insignificant for both materials. The observed increase of denture retention with time accentuates the impact of patient neuromuscular coordination developed with function.^38^

## Conclusion

Within the limitations of this study, the following conclusions could be reached:

BioHPP exhibited the highest hardness and flexural strength values compared to all groups of polyamide resin.

Modification of polyamide resin with either 2.5 or 5 wt% of Al_2_O_3_ NPs demonstrated a significant increase in its surface hardness and flexural strength values compared to the control group.

The higher concentration of Al_2_O_3_ NPs (5 wt%) seemed to significantly increase polyamide flexural strength values, with an insignificant increase in surface hardness values compared to the lower concentration (2.5 wt%).

Both 5 wt% Al_2_O_3_ NP-modified polyamide resin and BioHPP could be considered promising alternative denture base materials with satisfactory adaptation and retention.

## Authors’ Contributions

Both RME and RMA conceived and designed the work, collected the data, contributed to data analysis, and wrote the paper. RMA performed the in vitro part of the study, and RME performed the clinical part. Both authors contributed to the critical revision of the manuscript and read and approved the final version to be published.

## Acknowledgments

None.

## Funding

Self-funded.

## Competing Interests

The authors declare no conflict of interests related to the publication of this work.

## Ethics Approval

The study protocol was approved by the institutional ethical committee of the Faculty of Dentistry, Mansoura University, Egypt.
